# The effect of second language acquisition on central auditory processing abilities and its interaction with HIV

**DOI:** 10.3389/flang.2024.1427392

**Published:** 2024-08-14

**Authors:** Abby Kambhampaty, Christopher E. Niemczak, Samantha M. Leigh, Jonathan Lichtenstein, Monika Adhikari, Abigail M. Fellows, Albert Magohe, Jiang Gui, Linda Zhang, Enica R. Massawe, Jay C. Buckey

**Affiliations:** 1 Space Medicine Innovation Lab, Dartmouth College, Hanover, NH, United States; 2 Space Medicine Innovation Lab, Geisel School of Medicine at Dartmouth, Lebanon, NH, United States; 3 Department of Medicine, Dartmouth-Hitchcock Medical Center, Lebanon, NH, United States; 4 Department of Quantitative Biomedical Sciences, Geisel School of Medicine at Dartmouth, Hanover, NH, United States; 5 Department of Psychiatry, Dartmouth-Hitchcock Medical Center, Lebanon, NH, United States; 6 Department of Otorhinolaryngology, Muhimbili University of Health and Allied Sciences, Dar es Salaam, Tanzania; 7 Department of Obstetrics and Gynecology, Muhimbili University of Health and Allied Sciences, Dar es Salaam, Tanzania

**Keywords:** central auditory function, HIV, second language acquisition, Tanzania, multilingualism

## Abstract

**Introduction:**

Second language learning is a multifaceted task that benefits across numerous neurocognitive domains including central auditory processing. Existing cross-sectional and longitudinal data show that performance on tests of central auditory processing [central auditory tests (CATs)] worsens with HIV infection. Second language learning may modify this relationship. To explore the relationship between second language learning, central auditory processing, and its interaction with HIV, we assessed the effect of learning English as a second language on CATs among children both living with and without HIV (CLWH/CLWOH) in Dar es Salaam, Tanzania.

**Methods:**

Three hundred and seventy-two native Kiswahili speaking children aged 3–10 years old (196 CLWOH, 176 CLWH) were enrolled. Participants completed questionnaires about English language learning, socioeconomic status (SES), and health history. Three central auditory tests-the Triple Digit Test (TDT), the Staggered Spondaic Word Test (SSW), and the Hearing-In-Noise Test (HINT)-were used to assess each participant’s central auditory processing abilities. Multivariate linear regression was used to assess the effect of written and spoken English language learning at home and in school on CATs with age, HIV-status, and SES included in each model.

**Results:**

HIV status, age, and SES were all significant predictors of all three central auditory tests, with CLWH performing significantly worse on all three CATs than CLWOH. Children actively learning spoken and written English at home had significantly better central auditory processing abilities on the TDT compared to children not actively learning English at home (*p* < 0.01) independent of HIV status, age, and SES. Children learning spoken and written English at school performed significantly better on the HINT (*p* < 0.05) than those not actively learning English at school.

**Discussion:**

Learning English at home and learning English in school were associated with improved central auditory performance independent of HIV status, SES, and age. These findings also underscore the significance of second language acquisition as a potential mechanism of improving central auditory function within a Kiswahili-speaking cohort. This study found differences in central auditory processing between children exposed to English at home and in school, suggesting differences in language learning in both settings mediated by SES, and this benefit exists regardless of HIV status.

## Introduction

Tanzania is a multilingual society, with Kiswahili, commonly known as Swahili, as the country’s national language and over 120 spoken indigenous languages. English is taught at the secondary school level, and many children also learn English at home (Biswalo, 2022). Tanzania’s linguistic landscape is characterized by extensive multilingualism. While Kiswahili (a Bantu language) and English are considered high-status languages, it is important to acknowledge the presence of other major indigenous languages and language families, including Cushitic and Nilotic, in the country. Despite Tanzania’s multilingual nature, Kiswahili, originally spoken by a minority group and influenced by Arabic, was adopted as the official language after independence (1961), resulting in its widespread native use over time. Current educational policy supports Kiswahili as the language of instruction at the primary level and English at the secondary level, leaving other indigenous languages without support in the school system, which significantly impacts the multilingual students’ language acquisition and experiences (Roemer, 2024).

Previous research shows that children learning English as a second language show heightened auditory discrimination, enhanced attentional processing in the presence of background noise, elevated auditory pathway stimulation, and improved neural conduction in auditory pathways (Marques et al., 2021; Saito et al., 2022). The ability to perceive, understand, write, and speak a second language also engages a variety of neurocognitive domains because it requires fast and accurate processing, sustained attention, and good working memory (Morales et al., 2013; Kachlicka et al., 2019). Second language learning enhances cognitive flexibility, working memory, and processing speed (Morales et al., 2013; Kachlicka et al., 2019). In Dar es Salaam, Tanzania, the impact of English language acquisition as a second language on central auditory processing has not yet been investigated. This study examines how English language acquisition in a Kiswahili speaking cohort affects performance on three central auditory tests.

Many factors may affect the relationship between second language learning and central auditory processing. Previous research has shown that CLWH have diminished performance on central auditory tests compared to CLWOH (Niemczak C. et al., 2021). This study is particularly relevant to Dar es Salaam, where the overall HIV prevalence is about 6.3%, and the prevalence among children under 15 is 4.6% (Alexander Ishungisa et al., 2020; UNAIDS, 2021; Nyumayo et al., 2022). Previous studies have found a strong significant correlation between HIV positivity and low socioeconomic status in Dar es Salaam (Evans, 2002; Amuri et al., 2011). The relationship between second language acquisition and central auditory processing in a Kiswahili speaking cohort of CLWH and CLWOH has not been studied and may reveal whether HIV infection negates the beneficial effect of second language learning. Previous studies have found a correlation between low SES and poor central auditory abilities (Tabone et al., 2017) The aim of this exploratory study was to assess how English language acquisition among a Kiswahili speaking cohort impacts central auditory performance, while considering factors such as HIV status, age, and socioeconomic status.

We hypothesized that children who had started learning written and/or spoken English at home or in school would exhibit enhanced CAT performance. We further hypothesized that SES, age, and HIV would significantly affect CAT performance. Age has been shown to be a significant predictor of central auditory processing abilities, as previous research shows that children of lower age groups exhibit weaker performance on CATs (Niemczak C. et al., 2021). Nonetheless, we hypothesized that children learning English would exhibit enhanced CAT performance independent of age. Additionally, we expected that speaking a second language would correlate with better CAT performance compared to written language acquisition. Findings from this study could provide valuable information to health policymakers and educators in regions of high HIV prevalence by identifying the effect of second language learning on auditory processing in CLWH and CLWOH. Second language learning can improve auditory processing, which in turn can lead to better educational performance. Although the study suggests that while second language learning may improve auditory processing and thus educational outcomes, it does not necessarily protect against the direct effects of HIV. Instead, it might help mitigate some of the differences in educational performance that can arise due to the impact of HIV on cognitive functions.

## Methods

All research protocols were reviewed by the Institutional Review Boards of both Dartmouth College and the Muhimbili University of Health and Allied Science, and all research was completed in accordance with the Helsinki Declaration. The legal guardians of all participants provided written informed consent, and children participating provided assent.

This study used CAT measures and sociodemographic information from a cross-sectional sample derived from an ongoing longitudinal study in Dar es Salaam, Tanzania. Sociodemographic data regarding SES were obtained from the participants’ first visit. English language learning abilities and central auditory processing test scores were obtained from the participants’ most recent visit. All data were taken over the course of <5 years. Children came for CAT testing twice a year up to age 6 and then yearly. The mean number of visits was 3.6 for children living with HIV and 3.7 for children living without HIV. Participants included 372 children aged 3–10 years and included 196 CLWOH and 176 CLWH. Table 1 shows the demographic information of the study sample. Participants were excluded if they had a history of hearing loss, concussion, mental illness, chemotherapy, ear drainage, noise exposure, neurological disease, or any significant exposure to noise, chemicals, or gentamicin. Hearing loss was defined as an inability to hear at or below a 25 dB HL tone at any tested frequencies (0.5, 1.0, 2.0, and 4.0 kHz). These exclusions did not eliminate any participants. To determine HIV status, CLWOH need to have either one PCR test or a positive rapid test. CLWH needed to have a positive test in their medical record or test positive on a rapid test with subsequent confirmation via enzyme-linked immunosorbent assay (ELISA). All CLWH have had HIV since birth and were receiving antiretroviral therapy. Due to incomplete data, 32 children were excluded from the study. This included three children without an indicated HIV status, 14 children with unknown socioeconomic status, and the remaining children with missing information regarding second language learning status.

A sociodemographic questionnaire that inquired about English language learning, SES, age, parental status, and health history was administered to each participant. The questionnaire was derived from the Impaact P1104S Family Demographics and Socio-economic Questionnaire and was modified to better assess educational information (Boivin et al., 2018). Data regarding participants’ English language learning, including writing and speaking, were obtained from this questionnaire. In a section of the questionnaire titled “English Instruction”, participants were first asked a binary multiple-choice question: “Has the child started to learn spoken or written English at home or at school?”. If they answered *Yes*, they were asked to further specify their response by checking the appropriate option between: “learning written English at home”; “learning spoken English at home”; “learning written English at school”; and “learning spoken English at school”. Participants proceeded to answer the age that each of the listed instructions at home and school were started. Data from this questionnaire were stored in a Research Electronic Data Capture (REDCap) database.

To assess how central auditory function was influenced by SES, a questionnaire that inquired about income, father’s age, mother’s age, household water source, and electricity was administered. These data were stored in a REDCap database and used to form an SES composite variable. Specifically, a Principal Component Analysis (PCA) was employed to calculate socioeconomic status (Lichtenstein et al., 2022). PCA effectively reduced dimensionality of this covariate by transforming data into a set of uncorrelated principal components. The first principal component, which accounted for 32.7% of the variance, was used to calculate a single socioeconomic status score for each participant (Lichtenstein et al., 2022).

The CATs used for this study were the Triple Digit Test (TDT), Hearing-in-Noise Test (HINT), and the Staggered Spondaic Word Test (SSW). The mean age of completion for each test was 7.6, 7.5 and 6.9, respectively. Detailed information on the age ranges of completion for each test can be found in the Supplementary material. CATs were administered by Tanzanian colleagues in Kiswahili. In the TDT, recordings of three-digit triplets, such as 5-9-3 (spoken as tano-tisa-tatu in Kiswahili), were used as target stimuli. Kiswahili numbers from 1 to 9 have the same number of syllables. Triple digit recognition in the TDT was conducted in the presence of competing positive and negative Schroeder-phase masking noise (Niemczak C. et al., 2021). All three-digit-triplets were spoken by a male speaker. The TDT consisted of a total of 30 presentations of triple digits. The presentations were delivered with pairs of positive and negative-phase markers, presented in a random order for each pair. Each pair of maskers were presented at the same signal-to-noise ratio (SNR), ensuring consistent listening conditions. The test began with an initial SNR of 0 dBA, with the masked fixed at 65 dBA (Niemczak C. et al., 2021; Niemczak C. E. et al., 2021). Following each presentation or pair, the SNR was adjusted based on the participant’s performance. Incorrect digit reporting resulted in an increase of 2.0 dB in sound pressure level per missed digit; correct digits resulted in a target level decrease of 1.0 dB in sound pressure level. The speech reception threshold reflected the level of difficulty in recognizing the triplets. This value was determined based on the SNR of the last seven positive-phase presentations, and served as the primary indication of the participant’s ability to perceive and understand the digits in the presence of background noise (Niemczak C. et al., 2021).

The HINT, another speech-in-noise test, assessed subjects’ ability to perceive sentences in background noise administered in four localization conditions: noise front, noise right, noise left, and quiet. During each administration of the HINT, a distinct set of 20 sentences were presented in random order and delivered in the presence of speech-shaped energetic noise spectrally matched to the long-term average of all the HINT sentences (Niemczak C. et al., 2021). The signal-to-noise level was consistently set at 65 dBA, while the presentation level of each sentence was decreased if the previous sentence was repeated correctly, leading to a potentially more challenging listening condition. If the previous sentence was repeated incorrectly, the presentation level was increased to provide better audibility. This adaptive procedure allowed determination of the presentation level of each sentence on the list. The average presentation level of all the sentences defined the speech reception threshold for the test condition expressed as an SNR. A composite SNR of all three noise conditions was calculated (*(2 x noise front)* + *noise left* + *noise right*) and used as the primary variable for the HINT (Niemczak C. et al., 2021).

The Staggered Spondaic Word Test (SSW) assessed dichotic auditory processing of spondaic words presented to the listener. Different words were simultaneously presented to each ear in a staggered delivery (Amerman and Parnell, 1980; Katz and Smith, 1991). Each word used in the SSW consisted of two syllables and, during the test, the first word was presented to one ear with the second word presented to the other ear. The listener was tasked with repeating all the words they hear. Scoring is binary, with each word scored as either correct or incorrect. For example, considering two spondaic words, “upstairs” and “downtown”, the test presents these words in a sequence from the first to the third time point. At the second point, the words “stairs” and “down” are presented to different ears simultaneously. This test evaluates a person’s language processing ability and dichotic auditory abilities (Katz and Smith, 1991; Adams and Gathercole, 1995).

Data from CATs, the demographic questionnaire, and the SES PCA were analyzed and plotted using MATLAB^®^ 2020b. Multiple linear regression analyses, with individual CATs as the outcome variable and second language learning, SES, HIV status, and mean centered age as predictor variables were analyzed. Multiple models for second language learning at school and in the home were conducted on each CAT, with a Bonferroni correction for multiple comparisons. The estimates indicate the magnitude and direction of the effect of each independent variable on the dependent variable, in this case the CAT. The analysis of second language learning was divided into two main categories: children learning spoken and written English at **home** and children learning spoken and written English at **school**. This division allows for analysis of the two factors separately, as learning a second language at home may have socioeconomic implications and is a different structure than learning a second language at school, where regular assignments and social interactions allow for consistent progress and language exposure.

## Results

### Demographic analyses

Table 1 provides demographic and educational information for CLWH and CLWOH. No statistically significant difference was found in school attendance between the CLWH (89.8% attendance) and CLWOH (93.9% attendance) children. Of these groups, 58.5% of CLWH and 63.2% of CLWOH reported exposure to English. A high proportion of children reported learning either written or spoken English at school, which was more than double the number of children learning written or spoken English at home. Additionally, the average socioeconomic status of the CLWH group was lower than that of the CLWOH group. Detailed information about the age ranges of children completing each Central Auditory test can be found in the Supplementary material.

### English language learning

Table 2 presents the results of a linear regression analysis using Central Auditory Tests (CATs) as the dependent variables and “Exposure to English”—a composite variable that combines multiple English language learning variables related to speaking and writing—SES, HIV status, and age. Participants without exposure to English had an SNR on the TDT of 1.48 dB higher (i.e., poorer) than those with exposure to English (*p* = 0.001). This trend was also observed for SSW and HINT scores as well.

**FIGURE 1:**
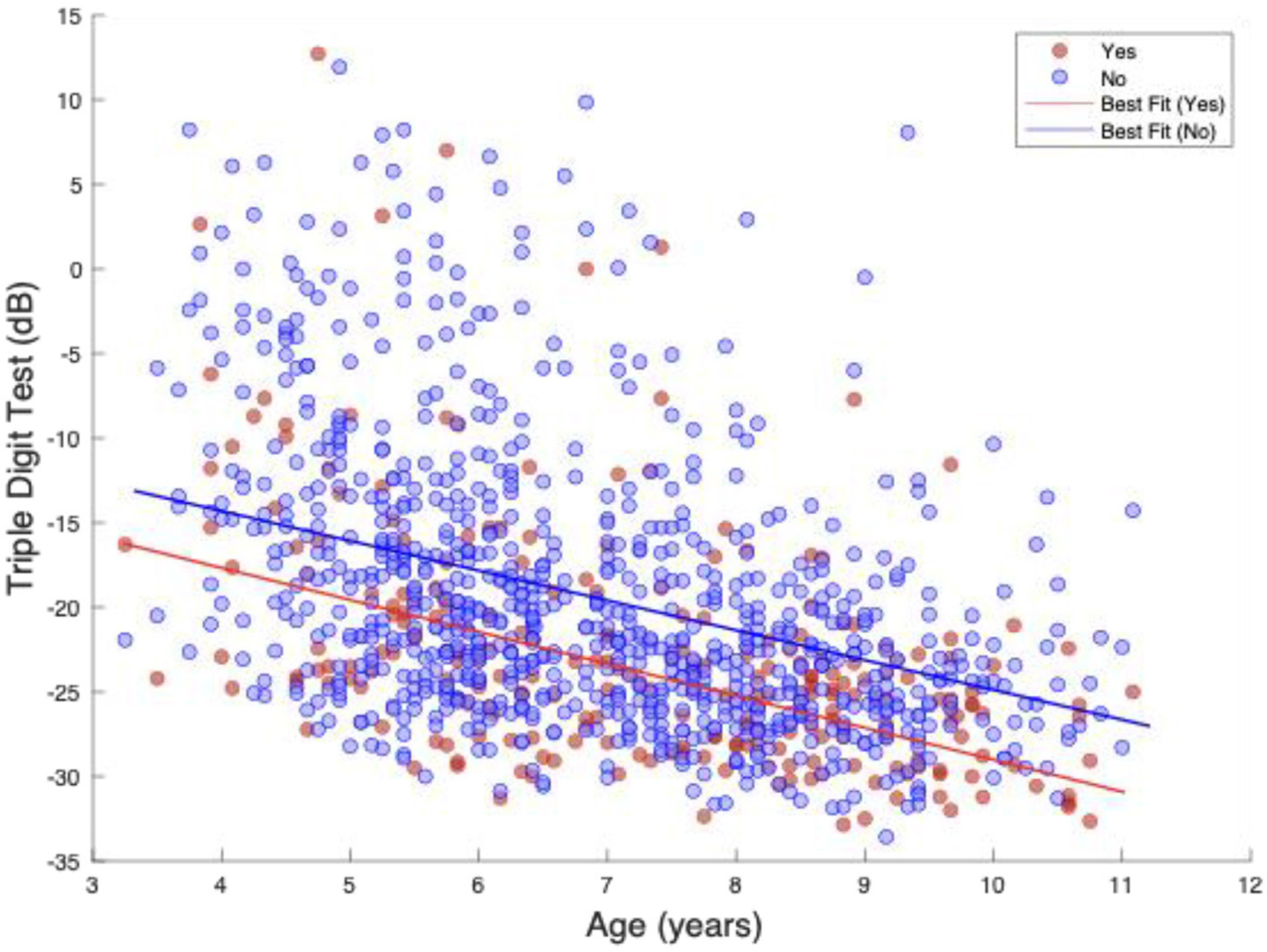
Triple Digit Test vs. written English home. This figure is a line plot showing the relationship of Triple Digit Test score (dB SNR) for Written English at Home categories over different age groups. Blue points represent the scores of individuals who indicated that they are not learning written English at home, and the red points represent the scores of individuals who indicated that they are learning written English at home. The lines represent lines of best fit, showing that the performance of children not learning written English at home is diminished in comparison to those who are.

Next, we assessed the effects of written and spoken English at home on central auditory processing abilities. Table 3 presents the effect of “Written English at Home”, or “Spoken English at Home”, along with socioeconomic status, HIV status, and age on the central auditory tests. Results from this table showed performance on the TDT to be significantly poorer in children not learning written or spoken English at home. Lower SES also correlated with poorer TDT scores. Coefficients suggest that not learning written or spoken English at home negatively affected performance on the SSW and HINT, but these values were not statistically significant. All three central auditory tests were significantly affected by HIV positivity and age. Figure 1 shows the relationship of Triple Digit Test score (dB SNR) for Written English at Home categories over different age groups.

**FIGURE 2:**
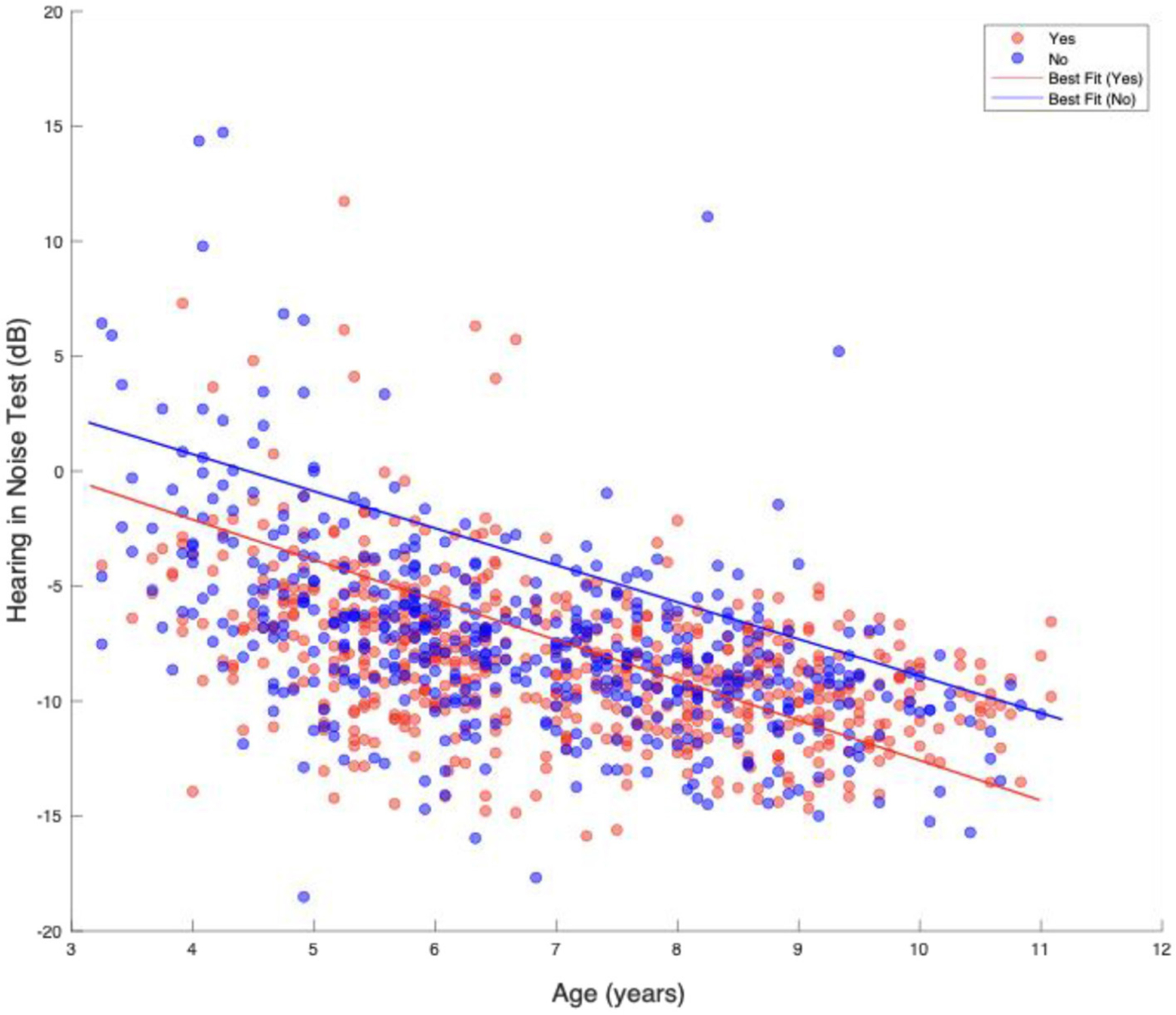
Relationship between HINT score and spoken English school. This figure is a line plot showing the relationship of HINT and age for spoken English at School categories over time. Blue points represent the scores of individuals who indicated that they are not learning spoken English at school, and the red points represent the scores of individuals who indicated that they are learning spoken English at school. The lines represent lines of best fit, showing that the performance of children not learning spoken English at school is diminished in comparison to those who are.

Next, we assessed the effect of written and spoken English at school. Table 4 shows the linear regression analysis using central auditory tests as the dependent variable and “Writing English at School” and “Spoken English at School” as independent variables, along with SES, age, and HIV status in each model. This revealed a high coefficient of 1.27 for the HINT for children not learning written English at school, which suggests that children without exposure to written or spoken English at school perform significantly worse on the HINT. This analysis also showed socioeconomic status to be a stronger predictor of TDT score than learning written and spoken English at school, with children from lower socioeconomic backgrounds having decreased performance on the TDT. All three central auditory tests displayed statistically significant HIV and age effects. Figure 2 shows a line plot of the relationships between HINT scores and age for the spoken English in school groups.

## Discussion

### English language acquisition and central auditory processing

Learning written and spoken English at home and in school is associated with better performance on central auditory tests in native Kiswahili speaking children. This suggests English language acquisition improves central auditory processing skills in Kiswahili speaking children independent of HIV status, age, and socioeconomic factors. This association was strongest between learning written and/or spoken English at home and better performance on TDT (*p* = 0.01). A strong relationship was observed between learning written and/or spoken English at school and the HINT (*p* = 0.03). HIV positivity and age played a significant role in each model, with poor central auditory performance in CLWH and children of younger ages. Diminished TDT performance also consistently displayed a significant correlation to low socioeconomic status. These findings support the hypothesis that English language learning enhances central auditory processing abilities in Kiswahili speaking children regardless of HIV status, and this correlation underscores the significance of language learning in understanding central auditory processing abilities within this Kiswahili speaking cohort.

Previous studies by Morales et al. (2013) have shown that second language learners have dynamic brains with increased cognitive flexibility, and these changes can occur during the earliest stages of second language acquisition. This type of cognitive flexibility enhances central auditory processing abilities and is most likely playing a role in the higher performance of children learning English seen in the current study. Bilingualism and second language learning has shown strong positive effects on working memory, which is particularly relevant to the HINT and SSW (Morales et al., 2013; Ware et al., 2021). Although the participants in the current study were not assessed for fluency, the cognitive benefits of bilingualism can be observed in children learning English as a second language (Adams and Gathercole, 1995). Children learning English as a second language often exhibit improved attention and concentration due to the cognitive demands of learning a new language at a young age (Kotz and Schwartze, 2010; Barac et al., 2014). Learning a second language, particularly English, involves exposure to a variety of speech sounds and nuances, and regular exposure to new speech in challenging listening conditions likely contribute to the observed relationship (Barac et al., 2014).

Previous research by Saito et al. (2022) delves into the relationship between auditory processing abilities and second language learning, particularly focusing on late Japanese-English bilinguals, highlighting that English language learners exhibit more precise discrimination abilities in distinguishing specific auditory dimensions. Studies done by Marques et al. (2021) offer compelling evidence regarding the influence of English language learning on cortical-level neural conduction of acoustic stimuli, finding significantly lower latency values in children undergoing English language instruction, indicating heightened auditory pathway stimulation and improved neural conduction (Specht, 2014; Marques et al., 2021). This study suggests that exposure to second language learning enhances attentional processing and the ability to suppress irrelevant sounds, demonstrating the impact of language acquisition on auditory processing (Marques et al., 2021).

The strongest correlation between English language learning and central auditory performance was seen with the TDT, which is a closed set task with a finite number of possible answers, numbers 1– 9, and consists of 30 presentations, demanding sustained attention for participants to succeed (Niemczak C. et al., 2021; Kwak et al., 2022). The discussed benefits of English language learning, including enhanced attentional processing, discrimination and cognitive flexibility, likely play a role in the observed association between second language learning and enhanced performance on the TDT, which requires highly accurate processing of auditory stimuli in the presence of competing background noise. Behaviorally, individuals learning a second language at home and school are exposed to multilingual auditory environments, forcing children to learn to perceive and process speech in noise, which is likely a cause of the enhanced digit recognition ability of English language learning children (Strydom et al., 2022).

### Differences in English exposure environment

Differences in this study were observed between children exposed to English at home and in school. Seventy-five participants indicated that they were learning written English at home and school. The differences in auditory processing based on English language exposure at home and school observed in this study can be attributed to the exposure environments. Learning English at home involves informal exposure at a younger age while learning English at school is more likely to be in chaotic, highly stimulating auditory environments (Strydom et al., 2022). The TDT exhibited stronger relationships to learning English at home than in school. Learning English at home involves early exposure to a new language’s auditory patterns and phonetics from a young age, which can improve individuals’ overall auditory processing skills and increase their ability to distinguish and recognize speech in sounds (Kachlicka et al., 2019; Strydom et al., 2022). According to studies performed on dual language learning children—defined as children exposed to two languages at home in early childhood— the processes of attention, selection, monitoring, inhibition, and flexibility constitute the core of children’s functional development (Barac et al., 2014). Studies done by Kousaie et al. (2019) using fMRI imaging to investigate how multilingual individuals perceive speech in noise showed that participants who learned non-native languages earlier in life showed a stronger ability to behaviorally process contextual information and greater neural recruitment in the left inferior frontal gyrus. Additionally, studies by Strydom et al. (2022) have determined the speech-in-noise perception of ESL speakers correlates significantly with years of exposure to the second language. Speech perception in noise was found to improve as the period of exposure to the second language increased, and therefore earlier exposure to the second language at home may be a potential reason for enhanced TDT performance (Strydom et al., 2022).

Improved HINT performance displayed a significant correlation to individuals learning English as a second language in school. The HINT requires listening to and repeating back entire sentences. Given the length of the sentence, understanding the context of the sentence is important for the participant to repeat it correctly (Nilsson et al., 1994; Niemczak C. et al., 2021).

The HINT presentation level is adjusted based on performance and includes a lengthy number of trials, so the HINT is a measure of adaptability to varying auditory environments and attention span (Nilsson et al., 1994; Novelli et al., 2018; Niemczak C. et al., 2021). Previous studies have found a strong correlation between HINT performance and age, which is supported by findings from the current study (Novelli et al., 2018; Kartal Özcan et al., 2023).

Strydom et al. (2022) delved into the effects of language learning on selective auditory attention and speech-in-noise perception among English Second Language (ESL) learners aged seven to 8 years. The majority of urban school listening environments are noisy and chaotic, and children must function in these environments every day, experiencing the pressure of having to perceive speech, in both their first and second language, in the presence of competing background noise (Strydom et al., 2022). Children learning English as a second language in school must perceive and process speech in such non-optimal listening environments, meaning these children often have experience with the skills tested by the HINT and TDT (Strydom et al., 2022). Additionally, previous research suggests that speech-in-noise perception in ESL speakers correlates with years of exposure to the second language, as earlier exposure to a second language is associated with improved understanding of speech-in-noise (Strydom et al., 2022). Age of exposure is influenced heavily by socioeconomic status and choice of school, with children from higher socioeconomic backgrounds often going to private schools which teach English at the primary school level in Tanzania (Jackson Nyamubi, 2019; Strydom et al., 2022; Biswalo, 2022). Therefore, it is likely that exposure to English in school, mediated by socioeconomic factors, enhances a student’s ability to focus in varying auditory environments, as supported by findings from the current study that show English language learning in school to be a strong predictor of both the TDT and HINT (Strydom et al., 2022; Biswalo, 2022).

Although the HINT and TDT are speech-in-noise tasks that use a SNR metric as the outcome variable, the TDT displayed a stronger correlation to English language learning, particularly at home. The HINT is a composite score of three spatial orientations of the speaker and background noise, and utilizes speech-shaped noise to match the long-term average of the presented sentences (Nilsson et al., 1994; Niemczak C. et al., 2021). Additionally, successful performance on the HINT requires repeating back entire sentences, leveraging contextual clues to help participants repeat the sentence correctly (Nilsson et al., 1994; Niemczak C. et al., 2021). The TDT requires remembering three unrelated numbers rather than long sentences. The precise reason for the differences between the tests is currently unclear, but highlights variability among speech-in-noise measures.

Results from the Staggered Spondaic Word Test (SSW), although not statistically significant, showed a pattern of children not learning English in school and home performing worse on the test. The SSW evaluates a participant’s ability to process language quickly and accurately identify words presented to each ear simultaneously, requiring high attention and processing speed (Amerman and Parnell, 1980; Keith, 1983; Katz and Smith, 1991). English language learning children are likely to be exposed to new and diverse auditory patterns, and develop stronger central auditory processing abilities, discrimination abilities in the presence of distraction such as dichotic noise, and working memory, and can therefore be expected to perform well on the SSW (Marques et al., 2021; Saito et al., 2022). Previous studies investigating the effects of English language acquisition age on auditory processing revealed that early English language learning children outperform monolingual peers in tasks assessing dichotic auditory processing, such as the Dichotic Digits test (Dastgerdi et al., 2023). The results highlight how early English language learning fosters superior binaural and temporal pattern processing, emphasizing broader cognitive benefits of second language learning in terms of dichotic processing (Dastgerdi et al., 2023). In the current study, it is unclear exactly why the association between SSW performance and English language learning was found to be insignificant. The SSW requires individuals to process the phonological components of the word, and the test requires a strong combination of auditory processing, memory, and cognitive demands, and therefore, it is possible that this central auditory measure is difficult for many children to complete.

### HIV, socioeconomic status, and central auditory processing

Previous studies have demonstrated the detrimental effects of HIV infection on central auditory processing, and results from the current study agree with these findings showing that CLWH perform more poorly on central auditory tests that CLWOH (Niemczak C. et al., 2021). Individuals living with HIV have differences in cortical function needed for central auditory processing, relating to gray matter atrophy, axonal injury, loss of axonal density, and diffuse white matter abnormalities in the internal capsule, thalamus, and corpus callosum (Kuhn et al., 2018; Zhan et al., 2018; Niemczak C. et al., 2021). Findings from the current study suggest that socioeconomic status is a significant predictor for TDT performance, with children from low socioeconomic backgrounds performing poorly on the TDT. This correlation appears to be the opposite for the HINT and SSW, and the reason for this finding is unclear. All three CATs were significantly affected by age, with older children performing better on CATs as expected. Given that findings from the current study show that second language learning enhances central auditory processing abilities, promoting second language learning can potentially contribute to improved central auditory performance in this specific population.

## Limitations

We recognize several limitations to this study. Previous research has demonstrated that age is an independent predictor of central auditory tests (Pichora-Fuller and Singh, 2006). Age-related changes were not the focus of the current study, but their influence on central auditory processing and English language learning were considered. We observed an age effect on central auditory test results; however, even with the age group included in the model, the central auditory tests were still significantly associated with English language learning. While the testing age range was −9, most participants that completed tests were aged 6–9. This limits the generalizability of the study’s findings to the entire specified age range. In this study, we only used the TDT, SSW, and HINT as the primary outcome variables. There are other central auditory tests and measures that can be used to determine the association between English language learning and central auditory processing. Further development of central auditory testing methods may produce a more sensitive test in measuring second language learning’s association to central auditory processing. The reliability and validity of each CAT in Kiswahili have not been formally assessed, however the CLWOH group provides a normative comparison group for this study. Also, these tests have been used in multiple of our cited studies and were developed in collaboration with Tanzanian colleagues.

Another limitation present in this study is that it does not address proficiency of English language learning, and only measures the relationship between children who have had exposure to English language learning and central auditory processing. Given that Kiswahili is spoken by over 90% of the population of Dar es Salaam, it was assumed that the children in this survey were native speakers of Kiswahili, and therefore English was their second language (Woodward et al., 2009). For future research, it is essential to consider the linguistic diversity of participants, especially in regions such as Dar es Salaam and other parts of Tanzania with many different indigenous languages spoken. Future studies should gather information about participants’ mother tongues, recognizing that while a national language like Kiswahili is widely spoken, many Tanzanians also speak indigenous languages as their first language. Investigating whether the benefits observed in learning English as a second language extend to Tanzanian children whose second language is Kiswahili would provide valuable insights into the impact of language typology on language acquisition. Examining the relationship between children who have learned English for longer periods of time and assessing fluency may prove longitudinal causal relationships, and this study only proves significant associations between English language learning and central auditory measures. Additionally, complementing these findings by examining these relationships in HIV-positive and negative individuals in populations outside of sub-Saharan Africa with other first and second languages will allow for a better understanding of the generalizability of our findings. Longitudinal examination and assessment of language fluency is needed to provide further insight into the relationships between central auditory processing and language learning. Additionally, while formal validity testing of individual questions on the questionnaire used to collect the data was not conducted, the questionnaire’s development involved rigorous collaboration with Tanzanian colleagues and experts to enhance cultural and contextual relevance.

## Conclusion

Children actively learning English at home and in school demonstrated enhanced central auditory processing abilities independent of age, socioeconomic status, and HIV status, indicating that English language acquisition improves central auditory processing abilities in Kiswahili speaking children. Strong relationships from both learning English at school and home were observed, and no significant difference between the two settings was observed. Written and spoken English are both correlated with similar auditory benefits. This correlation highlights the importance of language skills as a potential influencing factor on central auditory function in a Kiswahili speaking cohort and emphasizes the need to consider language abilities in the context of HIV infection.

## Supplementary Material

Supplementary Figure 1

## Figures and Tables

**TABLE 1:** Demographic and English language information by HIV status.

		HIV-positive (*N* = 176)	HIV-negative (*N* = 196)	*P*-value
Attended school *N* (%)		158 (89.8%)	184 (93.9%)	0.42
Learning to read Kiswahili *N* (%)		139 (79.0%)	175 (89.2%)	0.22
Average age *N* (SD)		6.8 (1.3)	6.3 (1.7)	0.87
Average peripheral hearing (HL)		8.17	8.21	0.83
Average socioeconomic status *N* (SD)		−0.1 (0.9)	0.0799 (1.0)	0.19
Learning English	Exposure to English *N* (%)	103 (58.5%)	124 (63.2%)	0.39
	Learning written English at home *N* (%)	34 (19.3%)	43 (21.9%)	0.33
	Learning spoken English at home *N* (%)	38 (21.6%)	49 (25.0%)	0.25
	Learning written English at school *N* (%)	94 (53.4%)	115 (58.7%)	0.31
	Learning spoken English at school *N* (%)	90 (55.1%)	121 (61.7%)	0.20

This table shows age, peripheral hearing, SES, school attendance, and language skills by HIV status. Average age and average SES are displayed with the standard deviation for each group in parentheses. “Attended School” refers to the number of children that have attended school at some point of the participant’s life. “Learning to Read Kiswahili” indicates participants who were actively learning to read in Kiswahili. The “Exposure to English” row shows the number of participants in each category with exposure to written or spoken English at home or school, and the same format follows for the remaining English learning columns.

**TABLE 2:** Linear regression model for central auditory tests (CATs) with predictors “exposure to English”, socioeconomic status, HIV, and age.

		Estimate	*t*-stat	*p*-value	Adjusted *R*^2^
Triple Digit Test	“Exposure to English” (no)	1.48	1.58	**0.01**	0.21
	HIV	1.95	2.17	**0.03**	
	Mean centered age	−2.38	−7.68	<**0.0001**	
	SES	−0.87	−1.94	**0.05**	
Staggered Spondaic Word Test	“Exposure to English” (no)	4.70	1.89	**0.01**	0.244
	HIV	6.18	3.4	<**0.0001**	
	Mean centered age	−5.61	−8.97	<**0.0001**	
	SES	1.61	1.74	0.183	
Hearing-in-Noise Test	“Exposure to English” (no)	0.77	1.61	**0.044**	0.233
	HIV	1.34	2.85	**0.02**	
	Mean centered age	−1.34	−9.05	<**0.0001**	
	SES	0.18	0.83	0.406	

This model displays the association between “Exposure to English” and the three CATs of focus (Model specification: central auditory test ∼ “Exposure to English” + SES + HIV status + mean centered age). Results from this table include Estimates, *T*-statistics, *P*-values, and Adjusted *R*^2^ values. These results suggest that children not learning spoken or written English as a second language are performing significantly worse on the TDT, HINT, and SSW. Bold values indicate statistical significance at alpha < 0.05.

**TABLE 3:** Linear regression model for central auditory tests (CATs) with predictors written/spoken English at home, socioeconomic status, HIV, and age.

		Estimate	*t*-stat	*p*-value	Adjusted *R*^2^
Triple Digit Test	Spoken English at Home (No)	1.12	0.97	**0.03**	0.216
	HIV positive	2.15	2.44	**0.01**	
	Mean centered age	−2.45	−8.0	<**0.0001**	
	SES	−0.97	−2.18	**0.03**	
Triple Digit Test	Written English at Home (No)	1.94	1.22	**0.01**	0.20
	HIV positive	2.17	2.46	**0.01**	
	Mean centered age	−2.44	−7.93	<**0.0001**	
	SES	−0.99	−2.22	**0.026**	
Staggered Spondaic Word Test	Spoken English at home (no)	0.46	0.19	0.84	0.23
	HIV positive	7.11	3.95	<**0.0001**	
	Mean centered age	−5.77	−9.21	<**0.0001**	
	SES	0.98	1.06	0.28	
Staggered Spondaic Word Test	Written English at home (no)	1.49	0.59	0.55	0.23
	HIV positive	7.04	3.92	<**0.0001**	
	Mean centered age	−5.74	−9.16	<**0.0001**	
	SES	1.07	1.16	0.24	
Hearing-in-Noise Test	Spoken English at home (no)	0.12	0.21	0.83	0.227
	HIV positive	1.45	3.22	<**0.001**	
	Mean centered age	−1.37	−9.42	<**0.0001**	
	SES	0.09	0.42	0.66	
Hearing-in-Noise Test	Written English at home (no)	0.80	1.27	0.204	0.23
	HIV positive	1.39	3.11	<**0.01**	
	Mean centered age	−1.36	−9.32	<**0.0001**	
	SES	0.15	0.68	0.49	

This model displays the association between learning written/spoken English at home and the three CATs of focus (Model specification: central auditory test ∼ Written/Spoken English at home + SES + HIV status + age). Results presented in this table suggest that children not learning spoken or written English as a second language at home and children coming from lower socioeconomic backgrounds are performing significantly worse on the TDT. Bold values indicate statistical significance at alpha < 0.05.

**TABLE 4:** Linear regression model for central auditory tests (CATs) with predictors written/spoken English at school, socioeconomic status, HIV, and age.

		Estimate	*t*-stat	*p*-value	Adjusted *R*^2^
Triple Digit Test	Spoken English at school (no)	1.02	1.09	**0.04**	0.207
	HIV positive	2.03	2.26	**0.02**	
	Mean centered age	−2.41	−7.77	<**0.0001**	
	SES	−0.96	−2.14	**0.03**	
Triple Digit Test	Written English at school (no)	1.00	1.07	0.28	0.19
	HIV positive	2.02	2.23	**0.02**	
	Mean centered age	−2.39	−7.67	<**0.0001**	
	SES	−0.97	−2.18	**0.02**	
Staggered Spondaic Word Test	Spoken English at school (no)	4.21	2.23	**0.02**	0.241
	HIV positive	6.33	3.49	<**0.0001**	
	Mean centered age	−5.59	−8.92	<**0.0001**	
	SES	1.48	1.61	0.11	
Staggered Spondaic Word Test	Written English at school (no)	4.85	2.54	0.01	0.245
	HIV positive	6.07	3.33	<**0.0001**	
	Mean centered age	−5.47	−8.66	<**0.0001**	
	SES	1.54	1.67	0.09	
Hearing-in-Noise Test	Spoken English at school (no)	0.82	0.47	**0.03**	0.234
	HIV positive	1.3	2.85	<**0.0001**	
	Mean centered age	−1.33	−8.99	<**0.0001**	
	SES	0.18	0.83	0.40	
Hearing-in-Noise Test	Written English at school (no)	1.13	2.37	**0.01**	0.24
	HIV positive	1.21	2.26	<**0.001**	
	Mean centered age	−1.29	−8.72	<**0.0001**	
	SES	0.21	0.95	0.34	

This model displays the association between learning written/spoken English at school and the three CATs of focus (Model specification: central auditory test ∼ Written/Spoken English at school + SES + HIV status + age). Results presented in this table suggest that children not learning spoken or written English as a second language at school and children coming from lower socioeconomic backgrounds are performing significantly worse on the HINT and TDT, respectively. Bold values indicate statistical significance at alpha < 0.05.

## Data Availability

The raw data supporting the conclusions of this article will be made available by the authors, without undue reservation.

## References

[R1] Adams A-M, and Gathercole SE (1995). Phonological working memory and speech production in preschool children. J. Speech Lang. Hear. Res 38, 403–414. doi: 10.1044/jshr.3802.403 7596106

[R2] Alexander Ishungisa M, Moen K, Leyna G, Makyao N, Ramadhan A, Lange T, (2020). HIV prevalence among men who have sex with men following the implementation of the HIV preventive guideline in Tanzania: respondent-driven sampling survey. BMJ Open 10:e036460. doi: 10.1136/bmjopen-2019-036460 PMC7537429 33020084

[R3] Amerman JD, and Parnell MM (1980). The staggered spondaic word test: a normative investigation of older adults. Ear Hear 1, 42–45. doi: 10.1097/00003446-198001000-00007 7390066

[R4] Amuri M, Mitchell S, Cockcroft A, and Andersson N (2011). Socioeconomic status and HIV/AIDS stigma in Tanzania. AIDS Care 23, 378–382. doi: 10.1080/09540121.2010.507739 21347901

[R5] Barac R, Bialystok E, Castro DC, and Sanchez M (2014). The cognitive development of young dual language learners: a critical review. Early Child. Res. Q 29, 699–714. doi: 10.1016/j.ecresq.2014.02.003 25284958 PMC4180217

[R6] Biswalo U (2022). The Interplay between Students’ First/home Languages and School Culture on Students’ Learning of English in Tanzania. Available at: https://jriiejournal.com/the-interplay-between-students-first-home-languages-and-school-culture-on-students-learning-of-english-in-tanzania/ (accessed April 28, 2024).

[R7] Boivin MJ, Barlow-Mosha L, Chernoff MC, Laughton B, Zimmer B, Joyce S, (2018). Neuropsychological performance in African children with HIV enrolled in a multisite antiretroviral clinical trial. AIDS 32, 189–204. doi: 10.1097/QAD.0000000000001683 29112069 PMC5736412

[R8] Dastgerdi ZH, Seifi H, and Vahabi M (2023). The effect of second language acquisition age (AOA) on auditory processing skills. Ind. J. Otolaryngol. Head Neck Surg 75, 3221–3227. doi: 10.1007/s12070-023-03978-w PMC10645661 37974891

[R9] Evans R (2002). Poverty, HIV, and barriers to education: street children’s experiences in Tanzania. Gender Dev 10, 51–62. doi: 10.1080/13552070215916

[R10] Jackson Nyamubi G (2019). Socio-economic status as determinants of students’ performance in english language in secondary schools in Tanzania. Educ. J 8:110. doi: 10.11648/j.edu.20190803.14

[R11] Kachlicka M, Saito K, and Tierney A (2019). Successful second language learning is tied to robust domain-general auditory processing and stable neural representation of sound. Brain Lang 192, 15–24. doi: 10.1016/j.bandl.2019.02.004 30831377

[R12] Kartal Özcan E, Çekiç S, Sennaroglu G, and Soli SD (2023). Development of the Turkish hearing in noise test for children. Coch. Implants Int 24, 235–242. doi: 10.1080/14670100.2023.2179753 36856533

[R13] Katz J, and Smith PS (1991). The staggered spondaic word test: a ten-minute look at the central nervous system through the ears. Ann. N. Y. Acad. Sci 620, 233–251. doi: 10.1111/j.1749-6632.1991.tb51587.x 2035945

[R14] Keith RW (1983). Interpretation of the Staggered Spondee Word (SSW) Test. Ear Hear 4, 287–292. doi: 10.1097/00003446-198311000-00005 6653932

[R15] Kotz SA, and Schwartze M (2010). Cortical speech processing unplugged: a timely subcortico-cortical framework. Trends Cogn. Sci 14, 392–399. doi: 10.1016/j.tics.2010.06.005 20655802

[R16] Kousaie S, Baum S, Phillips NA, Gracco V, Titone D, Chen J-K, (2019). Language learning experience and mastering the challenges of perceiving speech in noise. Brain Lang 196:104645. doi: 10.1016/j.bandl.2019.104645 31284145

[R17] Kuhn T, Kaufmann T, Doan NT, Westlye LT, Jones J, Nunez RA, (2018). An augmented aging process in brain white matter in HIV. Hum. Brain Mapp 39, 2532–2540. doi: 10.1002/hbm.24019 29488278 PMC5951745

[R18] Kwak C, Seo J-H, Oh Y, and Han W (2022). Efficacy of the digit-in-noise test: a systematic review and meta-analysis. J. Audiol. Otol 26, 10–21. doi: 10.7874/jao.2021.00416 34775699 PMC8755436

[R19] Lichtenstein J, Bowers C, Amato J, Niemczak C, Fellows A, Magohe A, (2022). Nonverbal cognitive assessment of children in Tanzania with and without HIV. Child Neuropsychol 28, 107–119. doi: 10.1080/09297049.2021.1957809 34315334 PMC8648945

[R20] Marques PM, Mattiazzi ÂL, Ferreira L, Oppitz SJ, and Biaggio EPV (2021). The effect of learning english on P300 in children. Int. Arch. Otorhinolaryngol 25, e284–e288. doi: 10.1055/s-0040-1710304 33968234 PMC8096498

[R21] Morales J, Calvo A, and Bialystok E (2013). Working memory development in monolingual and bilingual children. J. Exp. Child Psychol 114, 187–202. doi: 10.1016/j.jecp.2012.09.002 23059128 PMC3508395

[R22] Niemczak C, Fellows A, Lichtenstein J, White-Schwoch T, Magohe A, Gui J, (2021). Central auditory tests to track cognitive function in people with HIV: longitudinal cohort study. JMIR Form. Res 5:e26406. doi: 10.2196/26406 33470933 PMC7902183

[R23] Niemczak CE, Lichtenstein JD, Magohe A, Amato JT, Fellows AM, Gui J, (2021). The Relationship between central auditory tests and neurocognitive domains in adults living with HIV. Front. Neurosci 15:696513. doi: 10.3389/fnins.2021.696513 34658754 PMC8517794

[R24] Nilsson M, Soli SD, and Sullivan JA (1994). Development of the Hearing in Noise Test for the measurement of speech reception thresholds in quiet and in noise. J. Acoust. Soc. Am 95, 1085–1099. doi: 10.1121/1.408469 8132902

[R25] Novelli CL, Carvalho NGD, and Colella-Santos MF (2018). Hearing in Noise Test, HINT-Brazil, in normal-hearing children. Braz. J. Otorhinolaryngol 84, 360–367. doi: 10.1016/j.bjorl.2017.04.006 28549874 PMC9449241

[R26] Nyumayo S, Konje E, Kidenya B, Kapesa A, Hingi M, Wango N, (2022). Prevalence of HIV and associated risk factors among street-connected children in Mwanza City. PLoS ONE 17:e0271042. doi: 10.1371/journal.pone.0271042 36346792 PMC9642876

[R27] Pichora-Fuller MK, and Singh G (2006). Effects of age on auditory and cognitive processing: implications for hearing aid fitting and audiologic rehabilitation. Trends Amplif 10, 29–59. doi: 10.1177/108471380601000103 16528429 PMC4111543

[R28] Roemer AE (2024). Second language acquiescence of multilingual students in Tanzania. Lang. Educ 38, 269–285. doi: 10.1080/09500782.2023.2186792

[R29] Saito K, Kachlicka M, Suzukida Y, Petrova K, Lee BJ, and Tierney A (2022). Auditory precision hypothesis-L2: dimension-specific relationships between auditory processing and second language segmental learning. Cognition 229:105236. doi: 10.1016/j.cognition.2022.105236 36027789

[R30] Specht K (2014). Neuronal basis of speech comprehension. Hear. Res 307, 121–135. doi: 10.1016/j.heares.2013.09.011 24113115

[R31] Strydom L, Pottas L, Soer M, and Graham MA (2022). Effects of language experience on selective auditory attention and speech-in-noise perception among English second language learners: preliminary findings. Int. J. Pediatr. Otorhinolaryngol 154:111061. doi: 10.1016/j.ijporl.2022.111061 35149369

[R32] Tabone N, Said J, Bamiou D, and Grech H (2017). The Impact of Socioeconomic Status on Auditory Processing Skills in Maltese Children. Msida: Department of Communication Therapy, Faculty of Health Sciences, University of Malta, 10.

[R33] UNAIDS (2021). Tanzania UNAIDS Fact Sheet. Available at: https://www.usaid.gov/tanzania/fact-sheet/jun-13-2023-tanzania-hivaids-fact-sheet (accessed March 22, 2024).

[R34] Ware C, Dautricourt S, Gonneaud J, and Chételat G (2021). Does second language learning promote neuroplasticity in aging? A systematic review of cognitive and neuroimaging studies. Front. Aging Neurosci 13:706672. doi: 10.3389/fnagi.2021.706672 34867264 PMC8633567

[R35] Woodward M, Lindfords A-L, and Nagle L (2009). A Sociolinguistic Survey of the Nyiha and Nyika Language Communities in Tanzania, Zambia and Malawi. Summer Institute of Linguistics International Publications 1, 145. Abailable at: https://www.sil.org/resources/publications/entry/9168 (accessed April 20, 2024).

[R36] Zhan Y, Fellows AM, Qi T, Clavier OH, Soli SD, Shi X, (2018). Speech in noise perception as a marker of cognitive impairment in HIV infection. Ear Hear 39, 548–554. doi: 10.1097/AUD.0000000000000508 29112532 PMC5920702

