# Supplementary figures and images for: The effect of second language acquisition on central auditory processing abilities and its interaction with HIV
Source: Front Lang Sci. Author manuscript; Available in PMC 2025 Aug 29. (PMC12393694; doi:10.3389/flang.2024.1427392)

## Supplementary Figure 1

Histograms of Age Ranges for Completed Tests

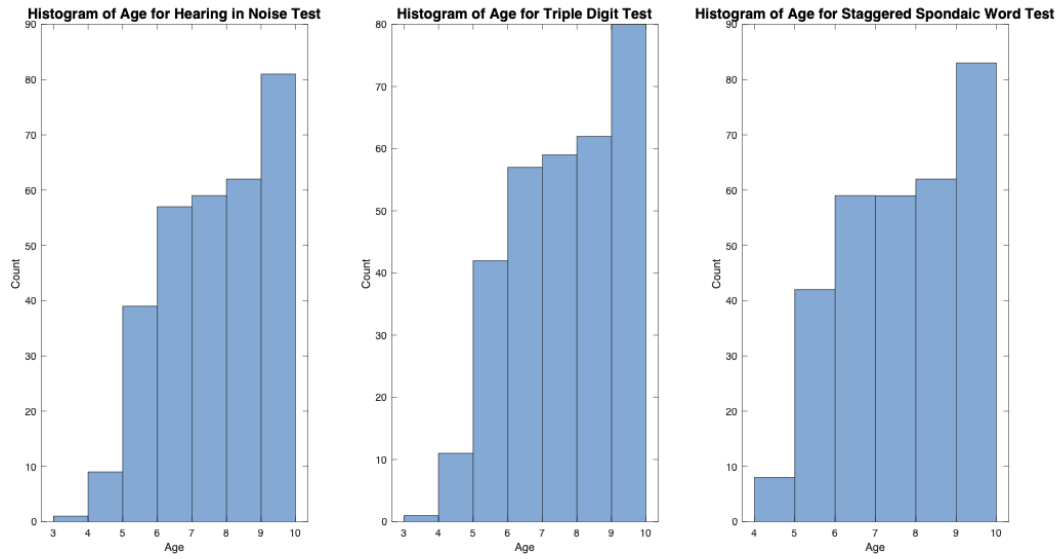

Supplement: Supplementary Figure 1 [file NIHMS2074880-supplement-Supplementary_Figure_1.pdf]
